# Purification of a Multidrug Resistance Transporter for Crystallization Studies

**DOI:** 10.3390/antibiotics4010113

**Published:** 2015-03-05

**Authors:** Kamela O. Alegre, Christopher J. Law

**Affiliations:** Institute for Global Food Security, School of Biological Sciences, Queen’s University Belfast, 97 Lisburn Road, Belfast BT9 7BL, UK; E-Mail: k.alegre@qub.ac.uk

**Keywords:** antiporter, antibiotics, crystals, membrane protein, transport, chromatography

## Abstract

Crystallization of integral membrane proteins is a challenging field and much effort has been invested in optimizing the overexpression and purification steps needed to obtain milligram amounts of pure, stable, monodisperse protein sample for crystallography studies. Our current work involves the structural and functional characterization of the *Escherichia coli* multidrug resistance transporter MdtM, a member of the major facilitator superfamily (MFS). Here we present a protocol for isolation of MdtM to increase yields of recombinant protein to the milligram quantities necessary for pursuit of structural studies using X-ray crystallography. Purification of MdtM was enhanced by introduction of an elongated His-tag, followed by identification and subsequent removal of chaperonin contamination. For crystallization trials of MdtM, detergent screening using size exclusion chromatography determined that decylmaltoside (DM) was the shortest-chain detergent that maintained the protein in a stable, monodispersed state. Crystallization trials of MdtM performed using the hanging-drop diffusion method with commercially available crystallization screens yielded 3D protein crystals under several different conditions. We contend that the purification protocol described here may be employed for production of high-quality protein of other multidrug efflux members of the MFS, a ubiquitous, physiologically and clinically important class of membrane transporters.

## 1. Introduction

Despite intense efforts put into the structural characterization of membrane proteins, their structures still represent only a small fraction of the total protein structures available (www.pdb.org). A comprehensive knowledge of the structure and mechanism of membrane proteins is especially relevant in the health and disease sectors, underscored by the fact that approximately 20%–30% of bacterial genomes are comprised of genes that encode membrane proteins, representatives of which contribute to the multidrug resistance profiles seen in bacteria today [1]. The relative scarcity of membrane protein structures can be attributed to hurdles in overexpression and purification, resulting in failure to obtain the milligram amounts of pure, stable protein needed for protein crystallography [2].

One of the most prolific, as well as physiologically and clinically important families of membrane proteins is the major facilitator superfamily (MFS). The MFS is the largest family of secondary active membrane transporter proteins, accounting for up to 1%–2% of prokaryotic genomes [3], with members that function in a diversity of transport roles in both prokaryotes and eukaryotes; these include nutrient, ion and even antibiotic uptake, and multidrug efflux [4]. To date, 3D structural data exist for only 14 unique members of the MFS; 11 of bacterial origin [5,6,7,8,9,10,11,12,13,14,15], one fungal [16], one plant [17] and one human [18]. Information derived from these structures has provided key insights into their mechanism and how they access, transport and release substrates. While one of these transporter structures is of EmrD, a multidrug efflux representative of the superfamily [7], the intermediate resolution of the structural model, combined with the absence of any bound antimicrobial substrate in the model, has prevented a more comprehensive understanding of the complete mechanism of multidrug efflux by this family of proteins. In consideration of the threat to human and animal health offered by the rise of multidrug resistance in pathogenic microorganisms, and to better understand drug efflux, there is need for more and higher resolution structural information on the membrane proteins that function in this process. A possible candidate for contributing to this information is the *Escherichia coli* multidrug efflux protein MdtM.

MdtM is a ~45 kDa member of the drug/H^+^ antiporter (DHA2) family subset of the MFS that encompasses a group of drug efflux proteins that contain 12 transmembrane spanning segments (TMS) [19]. Aside from its role in efflux of the antimicrobials chloramphenicol, ethidium bromide [20], and a range of quaternary ammonium compounds [21], recent studies revealed physiological roles for MdtM in alkaline pH homeostasis [22] and in bile salt resistance [23]. MdtM shares 41% sequence identity with MdfA [20], another MFS multidrug efflux protein from *E. coli*, and although extensive functional characterization has been done on both MdfA [24,25,26,27,28,29,30,31,32] and to a lesser extent on MdtM [20,21,22,23], the lack of structural data on either of these proteins has limited our ability to place these studies on the firm structural footing needed for a full understanding of multidrug efflux by members of the MFS.

Previous work performed in our laboratory found expression conditions which yielded about 0.15 mg of MdtM per litre of culture after purification [20]. MdtM was initially expressed as a hexahistidine (6-His) fusion protein and isolated by immobilized metal affinity chromatography (IMAC) and size-exclusion chromatography (SEC) in *n*-dodecyl β-d-maltoside (DDM) detergent solution. However, upon scale-up of the preparation, the protein sample quality was poor, with a pervasive protein contaminant, subsequently identified by the current work as a chaperonin, that co-eluted with MdtM in all the purification steps and confounded growth of protein crystals. We therefore revisited and modified the purification with the aim of producing MdtM protein of the quality and stability required for structural studies. Here we describe how addition of an extra four histidine residues to the metal affinity tag in combination with on-column removal of chaperonin contamination, followed by size exclusion chromatography produced a much purer protein that was more active with respect to substrate binding affinity, and more thermostable than that produced using the previous methodology. The isolated protein was monodisperse and stable in DDM detergent solution and was monomeric, as judged by a combination of SEC, dynamic light scattering (DLS) and blue native polyacrylamide gel electrophoresis (BN-PAGE) analyses. To maximize the probability of growing 3D crystals of MdtM, a detergent screen using a panel of seven detergents of differing alkyl chain lengths and/or micelle size was performed, and this screen identified the 10-alkyl carbon decylmaltoside (DM) as the shortest-chain detergent that could maintain MdtM in a stable state for crystallization trials. The 11-alkyl carbon detergent undecylmaltoside (UDM) was also effective at maintaining stability of the protein. Although MdtM retained ability to bind chloramphenicol in UDM and DM, the protein was significantly less thermostable in these detergents than in DDM. MdtM prepared using the modified protocol produced 3D protein crystals in DDM, but not in UDM or DM, under a number of different conditions.

## 2. Results and Discussion

Membrane protein overexpression is often the first hurdle in the process of obtaining the quantities of protein needed for crystallography studies. A protein overexpression screen performed in our laboratory had already identified LMG194 as the most suitable *E. coli* vector host for overproduction of MdtM [33]. LMG194 is often the default strain for hosting the pBAD expression vector due to its deficiency in arabinose metabolism [34] and has been used in the past for functional overexpression of other membrane transporters from pBAD (including the MFS glycerol 3-phosphate transporter GlpT, the γ-aminobutyric acid transporter GabP and the magnesium transporter CorA) in quantities sufficient for structural studies [3]. Although our previously published isolation was successful in overproducing the quantities of MdtM in *E. coli* strain LMG194 necessary for pursuit of biochemical and biophysical studies of the protein [20], subsequent scale-up of the purification resulted in a protein sample that was refractory to production of 3D crystals of MdtM for crystallographic studies. The inability of the protein to yield crystals was clearly a major obstacle for further understanding of the mechanism of multidrug efflux by members of the MFS. Therefore, to address this problem, we modified the isolation to improve the purity and stability of functional MdtM to enable the growth of 3D protein crystals.

### 2.1. Improved Purification of MdtM and Identification of Key Contaminant

**Figure 1:**
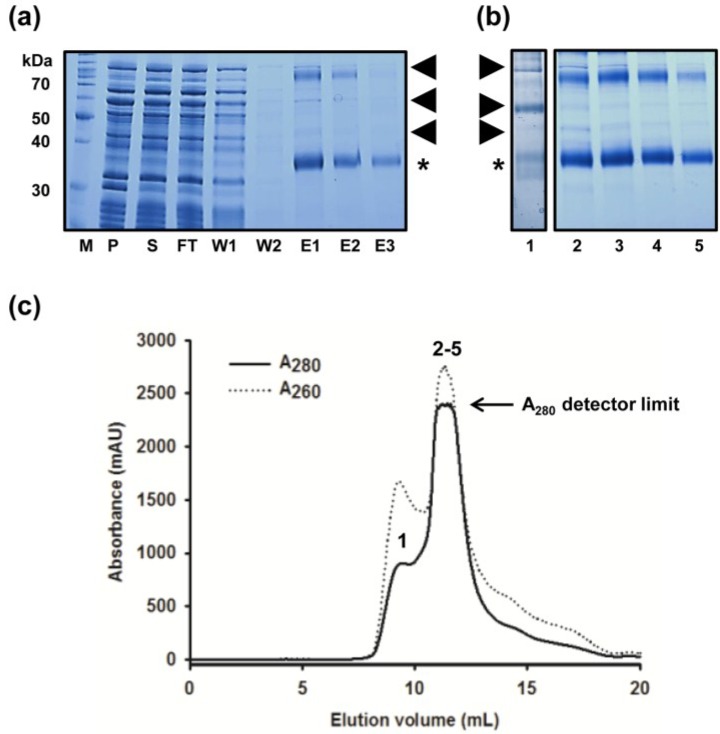
Purification of MdtM using the unmodified protocol. (**a**) Coomassie stained SDS-PAGE of protein fractions from Ni-NTA chromatography. Gel lanes were loaded with molecular weight marker (M), membrane pellet fraction (P), DDM-soluble fraction (S), column flow through (FT), wash one (W1), wash two (W2) and fractions eluted with 150 mM, 300 mM and 500 mM imidazole (E1, E2 and E3, respectively). Samples were incubated in SDS-PAGE sample loading buffer for 10 min at room temperature prior to loading onto the gel. A total of 8.4, 7.7 and 1.2 µg of protein were loaded from fractions E1, E2 and E3 respectively; (**b**) Coomassie stained SDS-PAGE of fractions from MdtM purification using FPLC size exclusion chromatography in 0.25 mM DDM. Lane 1, sample from the ~9.5 mL elution peak; lanes 2–5, samples from across the ~11.5 mL major elution peak. In both (**a**) and (**b**) MdtM is denoted with (*) and major contaminating proteins are labelled with arrows; (**c**) SEC chromatogram of MdtM. Protein absorbance at 280 nm and 260 nm is represented by the solid and dashed lines, respectively. Numerals above the absorbance peaks correspond to the gel lanes in panel (**b**).

Although the SEC chromatogram published in our original report on the purification of MdtM showed a single peak that was consistent with monomeric MdtM [20], scaling-up of the purification resulted in a poorly resolved SEC chromatogram (Figure 1c) with a large contaminant shoulder of A_280_~1000 milli Absorbance Units (mAU) at an elution volume of ~9.5 mL, and a major peak of >2300 mAU (corresponding to MdtM) that eluted at a volume of ~11.5 mL. Coomassie-stained SDS-PAGE analysis of the IMAC and SEC-purified MdtM protein fractions (Figure 1a,b, respectively) revealed the presence of at least three contaminating proteins with apparent molecular masses of ~40 kDa, ~60 kDa and ~95 kDa (in addition to the ~38 kDa and ~80 kDa bands that corresponded to monomeric and dimeric MdtM, respectively) that were likely candidates as confounding factors for production of 3D crystals of MdtM. It was clear, therefore, that the chromatography steps of the purification required optimization in order to improve the quality of the isolated transporter.

**Figure 2:**
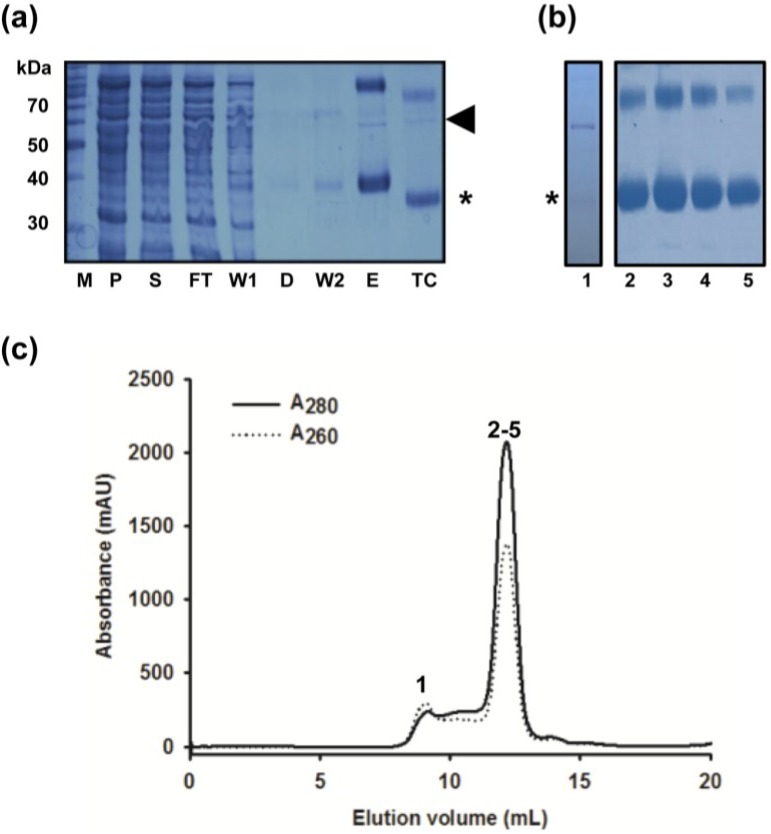
Purification of MdtM using the modified protocol. (**a**) Coomassie stained SDS-PAGE of protein fractions from Ni-NTA chromatography that included an incubation step with ATP to remove chaperonin contamination. Samples were incubated in SDS-PAGE sample loading buffer for 10 min at room temperature prior to loading onto the gel. Gel lanes were loaded with molecular weight marker protein marker (M), membrane pellet fraction (P), DDM-soluble fraction (S), column flow through (FT), wash one (W1), eluate from the ATP dissociation step (D), wash two (W2), 250 mM imidazole elution fraction (E), and the thrombin-cleaved fraction (TC). MdtM is denoted with (*) and chaperonin contaminant is marked with an arrow; (**b**) Coomassie stained SDS-PAGE of peak fractions from MdtM purification using FPLC size exclusion chromatography in 0.25 mM DDM. Lane 1, sample from the ~9 mL elution peak; lanes 2–5, samples from across the ~12 mL major elution peak. Lanes 2–5 were overloaded with sample to illustrate the absence of detectable 58 kDa contaminant; (**c**) SEC chromatogram of MdtM after removal of chaperonin. The protein absorbance at 280 nm and 260 nm is represented by solid and dashed lines, respectively. Numerals above the absorbance peaks correspond to the gel lanes in panel (**b**).

An initial step in this endeavour was facilitated by introduction of an elongated histidine tag into the MdtM construct. Four extra histidine residues were introduced by PCR amplification into the hexahistidine tag of the original MdtM construct to produce a decahistidine tag capable of tighter binding to Ni-NTA metal affinity resin. This permitted a more stringent wash protocol to be employed during IMAC by increasing the imidazole concentration in the wash buffer from the 30 mM concentration used in the original wash buffer to 50 mM, thereby providing an avenue for elution of unwanted proteins that could otherwise co-elute with MdtM at this stage of the purification. Although this resulted in a “cleaner” final eluate as assessed by SDS-PAGE (compare Figure 1a and Figure 2a), inspection of the SDS-PAGE gel still revealed a persistent contaminating band with an apparent molecular mass of approximately 58 kDa. Interestingly, the presence of an unidentified contaminating band of the same mass was noted before during purification of another MFS protein, the glycerol 3-phosphate transporter GlpT, that had been homologously overexpressed in *E. coli* LMG194 cells [35]. Therefore, in order to identify the contaminant, the 58 kDa band was excised from the gel, subjected to tryptic digest and the digest analysed by mass spectrometry. The analysis revealed that the band corresponded to the 57.3 kDa *E. coli* GroEL chaperonin protein (UniProtKB: P0A6F5). Just such a chaperonin contamination of the protein preparation would explain the 9.5 mL elution peak detected at 260 nm on the SEC chromatogram presented as Figure 1c; the A_260_:A_280_ ratio of this peak is >1, consistent with the presence of ATP in an ATP/chaperonin complex [36].

### 2.2. Removal of Chaperonin Contamination

Co-purification of chaperonin contaminants with the protein of interest is a common problem encountered during protein purification. A simple one-step incubation with ATP, however, can result in dissociation of chaperonins from the target protein and their subsequent removal [37]. Therefore, an ATP incubation step was introduced between the first and second IMAC column washes of the MdtM preparation to remove chaperonin contamination. After a first wash with 50 mM imidazole, a column containing decaHis-tagged MdtM bound to Ni-NTA resin was incubated with ATP dissociation buffer for 2 h at 4 °C. An additional wash of the column with 50 mM imidazole removed most of the dissociated chaperonin/ATP complex and residual ATP dissociation buffer from the column prior to elution of the decaHis-tagged MdtM with 250 mM imidazole and subsequent removal of the affinity tag by incubation of the sample with thrombin. Although SDS-PAGE analysis of the thrombin-cleaved sample (lane TC in Figure 2a) still indicated the presence of residual chaperonin, this was completely separated from MdtM in the downstream SEC purification step, the chromatogram of which (Figure 2c) revealed a major, well-resolved symmetrical peak at an elution volume of ~12 mL and a smaller peak at an elution volume of ~9 mL. In contrast to the SEC chromatogram of the hexaHis-tagged, non-ATP treated MdtM sample, the 260 nm trace corresponding to chaperonin decreased dramatically (compare Figure 1c and Figure 2c). Analysis of an overloaded SDS-PAGE gel (Figure 2b) revealed that the contaminating chaperonin was confined to the ~9 mL SEC elution peak (lane 1, Figure 2b) and that MdtM purity was upwards of 99% with no chaperonin present in the MdtM-containing SEC fractions (lanes 2–5, Figure 2b). The modifications to the isolation procedure resulted in about a 3-fold increase in protein yield, with ~2 mg of high-purity MdtM typically produced per 6 L of bacterial culture (~0.35 mg/L of culture), an amount compatible with initiating 3D structural studies of the protein.

### 2.3. DM Is the Shortest Alkyl-Chain Detergent that Maintains MdtM in a Stable and Monodispersed State

Growth of 3D protein crystals for use in X-ray crystallographic experiments requires the formation of protein-protein contacts between adjacent protein molecules in the crystal lattice and, commonly, the better those contacts the better-diffracting the crystals. In the case of membrane proteins the presence of the detergent molecules required to maintain these hydrophobic proteins in a soluble state in aqueous solution has a major influence on the extent of the protein-protein contacts formed [38]. The short-chain and small-micelle detergents that form smaller belts around the hydrophobic, transmembrane regions of membrane proteins can potentially facilitate formation of more contacts between exposed hydrophilic surfaces of the protein, and hence may permit growth of diffraction-quality crystals. Although generally there is correlation between better X-ray diffraction of membrane protein crystals with shorter chain detergents, these detergents can often prove denaturing. On the other hand, less denaturing detergents such as DDM form larger micelles that have the potential to hinder formation of protein-protein contacts and offer a challenge to production of structure-grade crystals. In consideration of the influence of detergent on the “crystallizability” of membrane proteins, it is often pertinent to choose the shortest-chain detergent that maintains the target protein in a stable, monodisperse state. Unfortunately, there is no “magic formula” for the choice of detergent, and identification of the most suitable one requires an empirical approach.

A previous investigation into the stability and monodispersity of MdtM in a panel of 12 detergents using small-scale SE-HPLC revealed that the protein was destabilized and tended to form aggregates in short-chain detergents such as octylglucoside, whereas longer chain detergents such as DDM maintained the transporter in a stable and monodisperse state [20]. To test if the same held true for MdtM purified using the modified, scaled-up protocol, we investigated the stability of MdtM in seven detergents, five each of different alkyl carbon chain length (OTG, NG, DM, UDM and DDM; 8, 9, 10, 11 and 12-alkyl carbon chain lengths, respectively) and two other detergents of 12-carbon alkyl chain lengths that form small micelles (LDAO and DDMG), using FPLC-SEC. Octylthioglucoside (OTG) was chosen as the shortest alkyl chain detergent to be tested because, in our hands, MdtM had a propensity to rapidly aggregate and precipitate out of solution in octylglucoside (OG).

**Figure 3:**
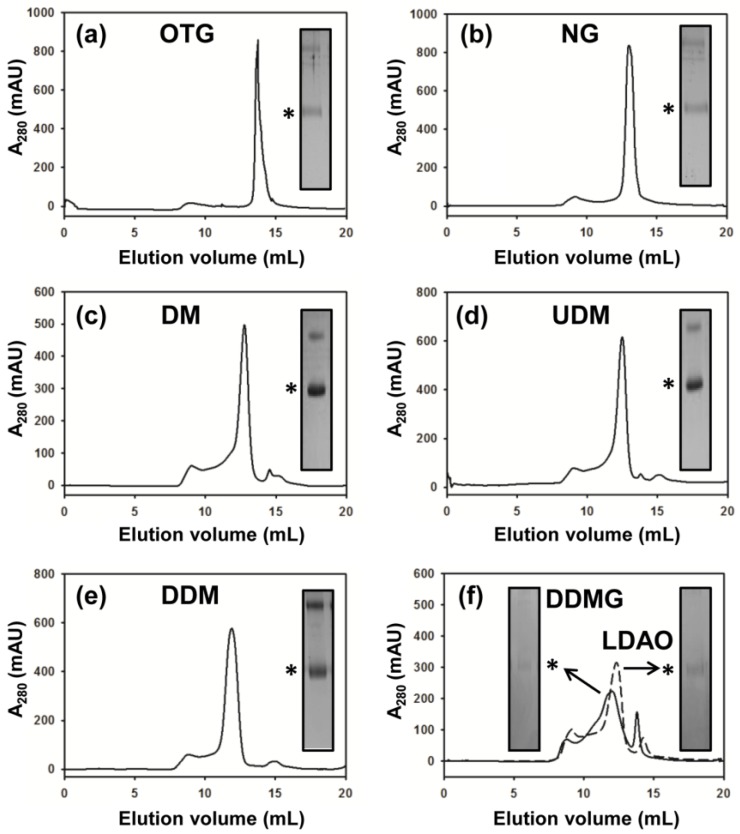
Detergent screening of MdtM. SEC chromatograms of MdtM in: (**a**) *n*-octyl-β-d-thioglucoside (OTG); (**b**) *n*-nonyl-β-d-glucoside (NG); (**c**) decylmaltoside (DM); (**d**) undecylmaltoside (UDM); (**e**) *n*-dodecyl β-d-maltoside (DDM); and (**f**) *n*-dodecyl-*N*,*N*-dimethylamine-*N*-oxide (LDAO) and *n*-dodecyl-*N*,*N*-dimethylglycine (DDMG). The insets of each figure represent gel slices from Coomassie stained SDS-PAGE analysis of the peak fraction from each run. Bands corresponding to monomeric MdtM are marked with an (*).

Thrombin-cleaved MdtM from the IMAC purification step was used for the detergent screen and samples were loaded onto a Superdex 200/30 GL column equilibrated with SEC buffer containing the detergent under test. The stability and monodispersity of MdtM in each of the detergents was assessed by analysis of the SEC chromatogram, and eluted peak protein fractions were further analysed by SDS PAGE. From the chromatograms presented in Figure 3 the MdtM protein-detergent complexes eluted at a volume that reflected the chain length of the detergent used in the SEC buffer. The protein appeared least stable in the small-micelle detergents LDAO and DDMG (Figure 3f, solid and dashed lines, respectively) as judged by the relatively poorly-resolved and low amplitude chromatogram peaks and, due to aggregation, protein samples from the peak SEC fractions gave rise to very weak bands in a Coomassie-stained SDS-PAGE gel (Figure 3f, insets). It was clear, therefore, that these small-micelle detergents lacked utility for subsequent crystallization trials of MdtM. In contrast, the protein appeared to be stable and monodisperse in the other detergents tested. In our detergent screen we saw an apparent improvement in MdtM stability, when compared with the previous study [20], in the 8-carbon alkyl chain detergent OTG (Figure 3a) and 9-carbon alkyl chain detergent NG (Figure 3b). However, the protein precipitated out of solution within minutes of elution from the SEC column in both these detergents and the weakly stained bands on SDS-PAGE (inset of Figure 3a,b) confirmed that significant protein aggregation had occurred; OTG and NG were therefore discounted as suitable detergents for MdtM crystallization screens. In contrast, the SEC behaviour of MdtM solubilized in the 10-alkyl carbon detergent DM (Figure 3c) and 11-alkyl carbon detergent UDM (Figure 3d)—in each case the protein eluted as a well-defined peak—suggested that both these detergents maintained the protein in a stable and monodisperse state, and the protein showed the same stability in the 12-alkyl carbon detergent DDM as in our previous study [20], with a symmetrical peak centred at an elution volume of ~11.5 mL (Figure 3e). The stability of MdtM in DM, UDM and DDM appeared to be confirmed by Coomassie-stained SDS-PAGE analysis (inset of Figure 3c–e, respectively), with intensely stained protein bands corresponding to monomeric and dimeric transporter visible on the gel. Therefore, to maximize the probability of growing diffraction-quality protein crystals, DM, UDM and DDM were chosen as the detergents for use in subsequent crystallization trials of the transporter.

### 2.4. Binding Affinity of Purified MdtM

**Figure 4:**
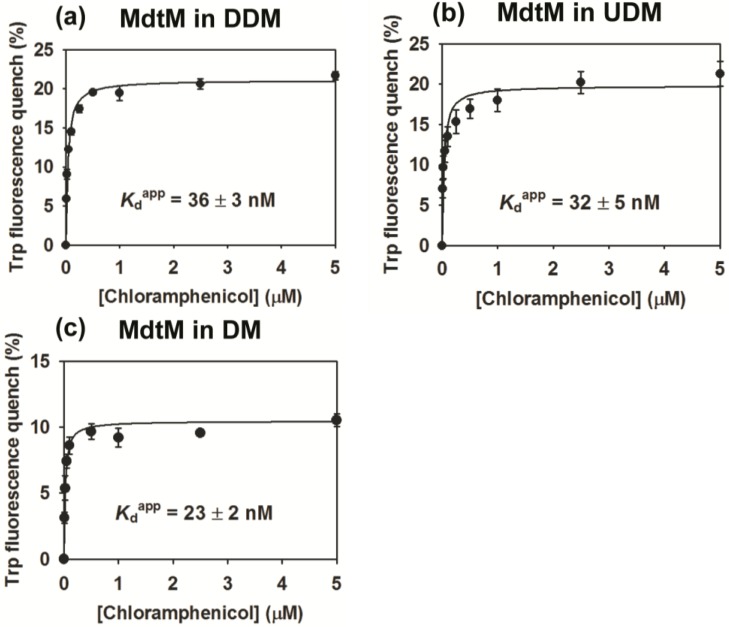
Substrate-binding affinity curves for chloramphenicol binding to MdtM in different detergents. (**a**) Binding affinity of chloramphenicol for MdtM in DDM; (**b**) Binding of affinity of chloramphenicol for MdtM in UDM; (**c**) Binding affinity of chloramphenicol for MdtM in DM. Data points and error bars represent the mean ± SD of three individual measurements.

To test for functional integrity of purified, decaHis tag-cleaved MdtM, its activity in DDM, UDM and DM-detergent solutions was assessed by exploiting the quenching of intrinsic tryptophan fluorescence of the protein upon binding of the antibiotic substrate chloramphenicol. For MdtM in DDM, the data fitted well to a single-site binding equation yielding an apparent binding dissociation constant (*K*_d_^app^) of 36 ± 3 nM (Figure 4a). This affinity for chloramphenicol is about 5-fold tighter than the previously reported *K*_d_^app^ of 170 ± 40 nM that was measured for MdtM purified in DDM using a protocol that did not include removal of contaminating chaperonin [20], and again highlights the efficacy of the modified purification for production of high-quality protein. The binding affinity of MdtM solubilized in UDM for chloramphenicol was very similar to that of the protein in DDM, with a measured *K*_d_^app^ value of 32 ± 5 nM (Figure 4b). The binding activity of MdtM was significantly greater when the protein was solubilized in DM, with a measured *K*_d_^app^ of 23 ± 2 nM (Figure 4c), and this may reflect binding of the antibiotic substrate to a different conformational state of the transporter in that particular detergent. The observation that substrate-binding affinity of MdtM was not only retained but also enhanced in the shorter alkyl chain DM detergent was encouraging for use of this detergent in subsequent crystallization trials of the protein.

The low nanomolar affinity of MdtM for chloramphenicol substrate is much tighter than the affinities reported for other multidrug transporters of the MFS for their substrates; purified MdfA typically binds substrates with affinity in the low μM range [29] and LmrP reconstituted into proteoliposomes binds Hoechst 33342 with an apparent *K*_d_ of 1.3 μM [39]. The relatively tight binding of MdtM to chloramphenicol suggests the protein may function as a high-affinity chloramphenicol transporter in *E. coli*.

### 2.5. Determination of the Oligomeric State of MdtM

**Figure 5:**
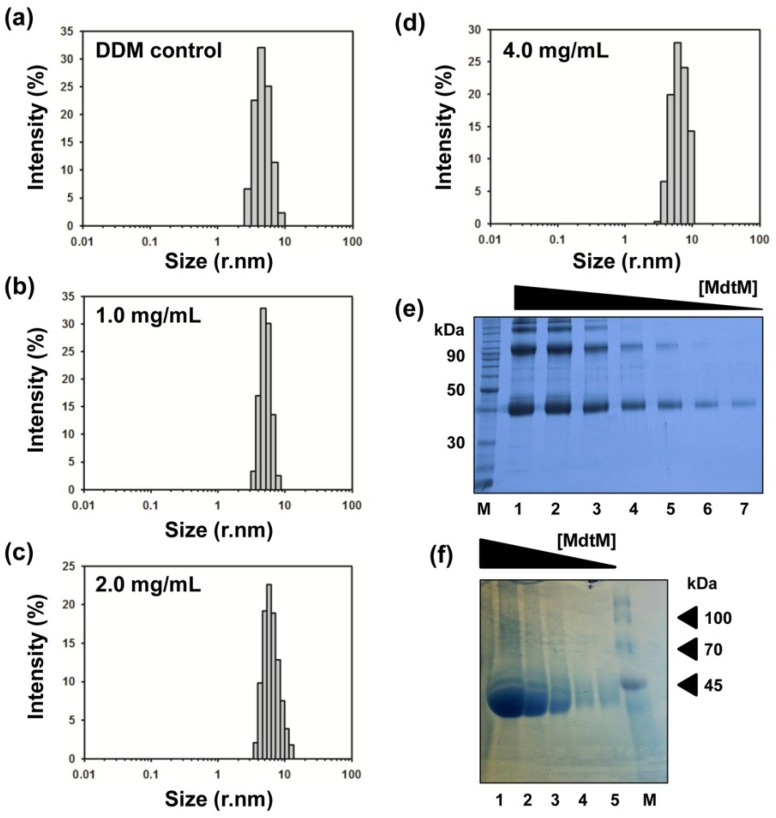
Oligomeric state of MdtM*.* Size distribution by intensity of (**a**) DDM-buffer solution alone, and (**b**) 1.0 mg/mL; (**c**) 2.0 mg/mL; and (**d**) 4.0 mg/mL of MdtM-DDM protein-detergent complex solution as determined by dynamic light scattering measurements; (**e**) Concentration dependent oligomerization of MdtM on SDS-PAGE. Lanes 1–7 contained 16 µg, 8 µg, 4 µg, 2 µg, 1 µg, 0.5 µg and 0.25 µg of MdtM protein, respectively. The lane labelled (M) contained molecular weight marker. Samples were run on a 15% SDS-PAGE gel and Coomassie stained. (**f**) Blue native PAGE of purified MdtM. Lanes 1–5 contained 33 µg, 11 µg, 3.7 µg, 1.2 µg and 0.4 µg of purified protein, respectively. The lane labelled (M) contained molecular weight marker. Samples were run on a BN-PAGE gradient gel of 3%–18% acrylamide and visualized by silver staining.

Although the closely-related MFS multidrug transporter MdfA was concluded to be monomeric in DDM solution [40], and previous work by our laboratory suggested MdtM is also a monomer in the same detergent [20], the extremely hydrophobic MdtM protein has propensity to form higher order oligomers on SDS-PAGE; intense bands corresponding to dimeric MdtM are readily apparent on SDS-PAGE gels containing protein samples from both the IMAC and SEC stages of the purification (see Figure 1 and Figure 2), and this raises questions as to the true oligomeric state of the isolated protein. The clear concentration dependence of MdtM oligomer formation under SDS-PAGE conditions is highlighted by Figure 5e; in addition to the band at ~38 kDa that represents the MdtM monomer, three intensely stained bands, corresponding to higher order oligomers (dimers, trimers and tetramers) of the protein that migrated with apparent molecular masses of approximately 90, 130 and 200 kDa, were readily visible in the gel lanes loaded with the highest concentrations of MdtM. However, as the MdtM concentration decreased there was a concomitant decrease in the presence of oligomeric forms of the protein until, at the lowest MdtM concentrations tested, the monomer was the only detected species. In consideration of the role that protein sample homogeneity plays in the growth of diffraction-quality crystals, the oligomeric state of MdtM in DDM, UDM and DM detergent solutions was estimated by dynamic light scattering (DLS) experiments. Such light scattering experiments can be used as a complementary tool to characterize the aggregation state of a protein sample [41], and DLS has been used before to assess the size both of maltoside-based detergent micelles [42,43,44] and membrane protein-detergent complexes [45,46,47,48].

**Figure A1:**
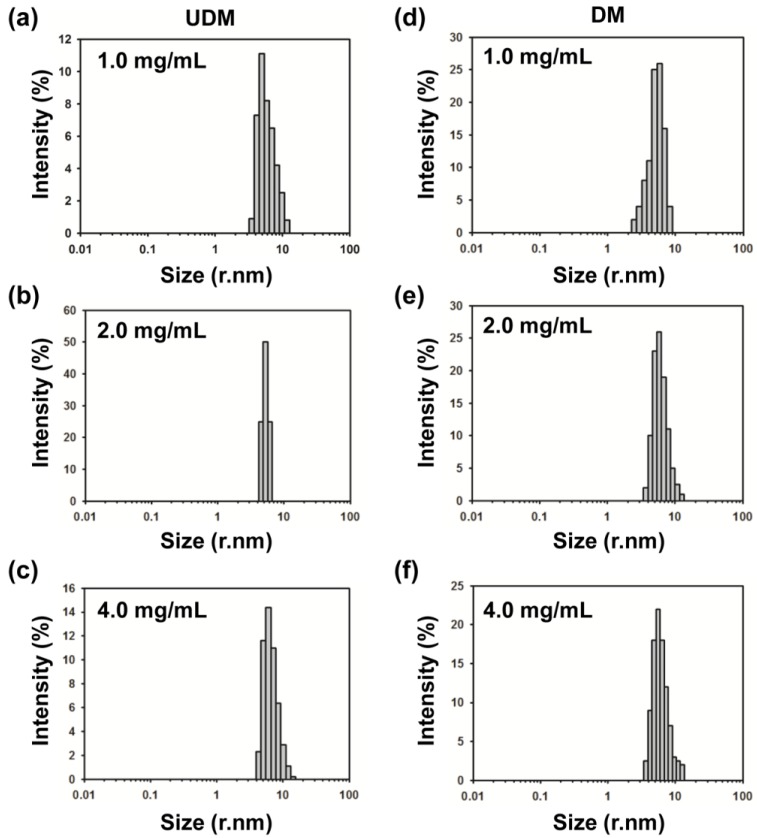
Oligomeric state of MdtM in UDM and DM detergents*.* Size distribution by intensity of 1.0 mg/mL, 2.0 mg/mL, and 4.0 mg/mL of (**a–c**) MdtM-UDM, and (**d–f**) MdtM-DM protein-detergent complex solutions as determined by dynamic light scattering measurements.

For DLS measurements, SEC-purified MdtM was diluted to give final protein concentrations of 1.0, 2.0 and 4.0 mg/mL in SEC buffer containing either 0.25 mM DDM, 0.6 mM UDM or 1.8 mM DM. DLS characterization of the negative control DDM detergent-buffer system (Figure 5a) showed that the DDM micelles scattered light effectively and gave rise to an intense scattering signal that corresponded to particles with a radius of about 4.5 nm, a value which is in general accord with radii measurements for DDM micelles determined by others using DLS [42,43,45] and small-angle X-ray scattering [44]. DLS analysis of the 1.0 mg/mL MdtM-DDM solution also revealed a heterogeneous intensity signal that represented scattering by particles with radii of between about 4–6 nm, with the most intense scattering signal emanating from particles with a radius of ~5 nm, similar in size to that of the DDM micelles alone. The results from the DLS experiments performed in DDM revealed that increasing the protein concentration to 2.0 mg/mL and 4.0 mg/mL did not significantly affect the estimated particle size of the MdtM-DDM protein-detergent complexes (Figure 5d,e, respectively); the most intense scattering signal in both samples emanated from particles with a radius of ~5.5 nm. Similar particle sizes were estimated from DLS measurements of MdtM in UDM and DM detergent solutions (Appendix Figure A1). The small size differences between detergent micelles alone and the protein-detergent complexes suggested that MdtM is monomeric in the detergents tested. Indeed, a particle radius of between 4–6 nm is a reasonable estimate for monomeric MdtM protein in a DDM detergent micelle and is comparable to the experimentally determined radii of other 12 TMS MFS transporters such as GLUT1 (5.0 nm) [49] and GlpT (4.6 nm) [35] that are monomeric in DDM.

To further support our contention that isolated MdtM is a monomer in detergent solution, the concentration-dependent behaviour of the protein in DDM was analysed by BN-PAGE (Figure 5f). BN-PAGE, which analyses proteins based on their native state, supported the results of the DLS analysis. Inspection of the BN-PAGE gel (Figure 5f) revealed that although monomeric MdtM comprised the dominant species (>90%) at all the protein concentrations tested, faint higher molecular weight bands were apparent in the lanes that contained the more concentrated protein samples. However, rather than being representative of dimeric or higher order MdtM, these high molecular weight bands could be attributed to increasing amounts of DDM or dye binding to MdtM, which could cause the protein to migrate in the gel with a higher apparent molecular mass [50]. The fact that MdtM runs predominantly as a single species on BN-PAGE over a range of protein concentrations suggests that it exists primarily as a monomer, and the oligomerization state of MdtM as determined by SDS-PAGE is not representative of the physiologically-relevant state of the protein or the state of the purified protein in DDM detergent solution.

### 2.6. Thermostability of MdtM in DDM, UDM and DM

**Figure 6:**
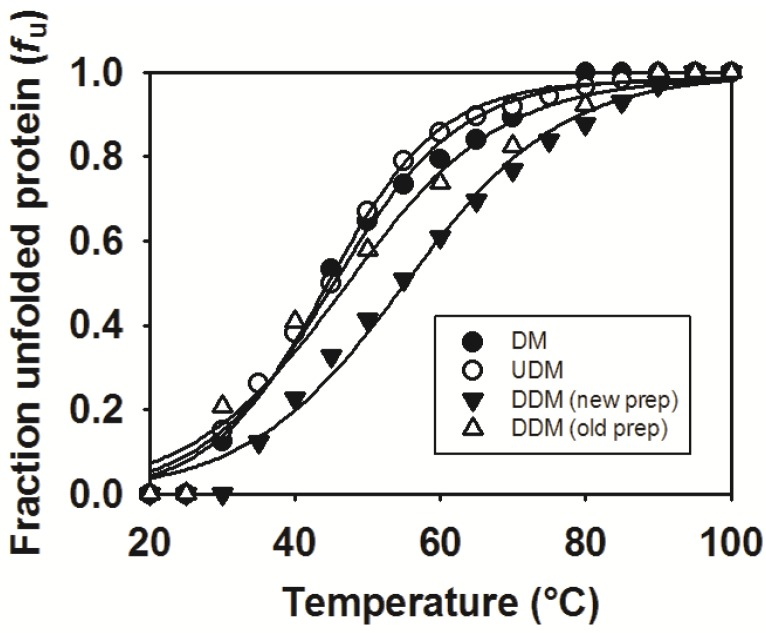
Thermal stability of MdtM in three different detergents represented as fraction of MdtM unfolded as a function of temperature. MdtM purified in DDM using the unmodified protocol is represented by open triangles. MdtM purified in DDM using the modified protocol is represented by filled inverted triangles. MdtM in UDM and MdtM in DM are represented by open and filled circles, respectively. Data points represent the mean of three individual measurements.

The stability of any given protein is an important variable that can influence crystallizability of that protein. Often, the protein of interest must be stable over a period of days to weeks and even months during the screening of crystallization space. Therefore, information on the conditions that promote sample stability is of value when performing protein crystallization experiments. Although finding the detergent conditions that yield the highest protein stability is of critical importance to successful membrane protein crystallization [51], another important crystallization variable is the thermal stability of the protein. We therefore measured and compared the thermostability of DDM-solubilized MdtM purified using the previously published protocol [20] with that of the protein purified using our modified one. This comparison revealed that MdtM prepared using the latter method had a melting temperature (*T*_m_) of 55 ± 1.0 °C, whereas the *T*_m_ of MdtM purified using the former method was 47 ± 1.5 °C (Figure 6). This represented an increase of ~8 °C in the thermostability of MdtM prepared using the modified purification protocol, and indicated that this protein may be more amenable to crystallization in three dimensions. Protein purified in DDM using the modified protocol was also significantly more thermostable than protein purified using the shorter alkyl chain detergents UDM and DM; in UDM the *T*_m_ of MdtM was 44 ± 0.6 °C and in DM the *T*_m_ was 45 ± 0.8 °C. The observed decrease in thermostability in UDM and DM may be a consequence of delipidation of the protein by these shorter alkyl chain detergents and it is possible, therefore, that MdtM requires interaction with a particular amount and/or type of phospholipid for preservation of stability [52].

### 2.7. Crystallization of MdtM

**Figure 7:**
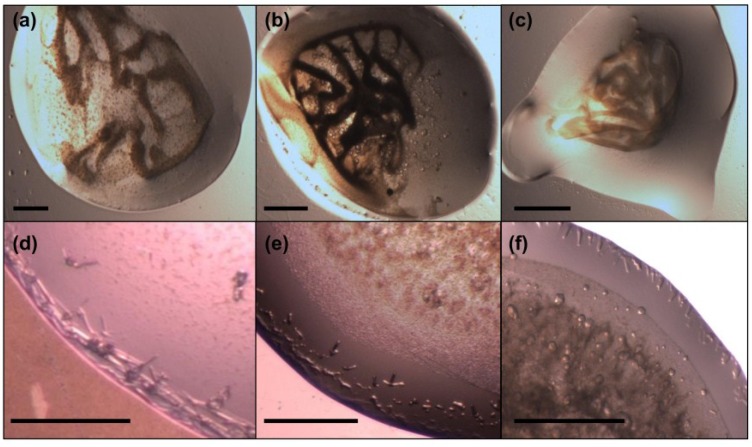
MdtM crystallization trials*.* Images in panels (**a**–**c**) recorded the results of crystallization trials performed with MdtM purified using the original, unoptimized protocol and crystallization buffers contained in the commercially available MemGold2 screening kit (Molecular Dimensions). Images in panels (**d**–**f**) represent the results of crystallization trials performed using the same conditions as those in (**a**–**c**), respectively, but with MdtM protein purified using the optimized purification protocol. Full details of the crystallization conditions are described in the text. Scale bars in (**a**–**c**) represent ~500 μm. Scale bars in (**d**–**f**) represent ~200 μm.

Crystallization trials of MdtM purified using the previously published protocol [20] were performed by screening protein samples against various commercial screens that contained a variety of different polyethylene glycols (PEGs), salts and buffers. Screening of over 1400 unique conditions yielded no positive hits, although many of the conditions showed amorphous precipitation or phase separation, and yet others contained DDM detergent crystals (Figure 7, panels a–c). The same screen was repeated for protein isolated using the modified purification protocol. To address the problem of detergent crystal formation, the concentrations of DDM in the IMAC and SEC chromatography buffers were reduced stepwise from 2.0 mM to 0.25 mM, and subsequent removal of excess DDM carried over from each chromatography step was performed by implementation of a centrifugal filtration step using a Centricon device (Millipore, Livingston, UK) with a 100 kDa MWCO, which also mitigated concentration of “empty” DDM micelles during protein concentration. The improvement in protein sample quality offered by the modified purification protocol led to a number of crystallization conditions that yielded positive hits and a significant reduction in the number of conditions containing DDM detergent crystals. The most promising hits were obtained using low molecular weight PEG crystallization buffers from the MemGold2 kit (Molecular Dimensions, Newmarket, UK). Figure 7 compares the results of just three of these crystallization trials performed with MdtM purified using the previously published, unoptimized protocol (panels a–c) and the same protein prepared using the optimized purification (panels d–f). Initial crystallization trials using unoptimized MdtM and a precipitant consisting of 0.2 M NaCl, 29% v/v PEG 400 and 0.05 M calcium acetate, pH 5.0, resulted in formation of a dark brown precipitate (Figure 7a). After two weeks, irregular crystals, probably of DDM detergent, formed in the centre of the drop. The same precipitant and incubation conditions applied to MdtM purified using the modified purification yielded a shower of fine needles with dimensions of ~50 μm × ~8 μm at the vapour/liquid interface on the edge of the drop (Figure 7d). These crystals continued to appear for a further 2 to 3 weeks. Similar results were obtained for the crystallization trials that used a precipitant consisting of 0.2 M sodium acetate, 28% v/v PEG 400 and 0.1 M MES, pH 6.5 (Figure 7b,e). Use of protein prepared using the unmodified protocol with these conditions resulted in formation of a light brown precipitate after two days of incubation at 18 °C, followed by formation of an amorphous precipitate a week later (Figure 7b). The precipitates in this drop presumably contained denatured protein. After modification of the MdtM purification, a fine precipitate formed in the bottom of the drop after five days, and this was followed eight days later by the appearance of 3D, matchstick-like protein crystals with dimensions of ~50 μm × ~10 μm × ~10 μm that formed around the edges of the drop (Figure 7e). The final condition that gave a positive hit contained 0.3 M barium chloride, 34% v/v PEG 400 and 0.1 M MES, pH 6.0, as the precipitant (Figure 7c,f). Under these conditions, MdtM purified using the unmodified protocol denatured in the drop after two days, resulting in formation of a light brown precipitate (Figure 7c). In contrast, the same crystallization conditions using MdtM from the modified purification produced flat needles, with dimensions of ~40 μm × ~10 μm, that grew around of the edges of the whole drop within seven days and continued to grow for a further two weeks (Figure 7f). Thin needles also appeared in the centre of the drop after one week. In all cases the crystals stained up with a 0.05% w/v solution of methylene blue, confirming they were protein and not salt.

Intriguingly, no crystal hits were observed when protein was purified in UDM or DM; this was unexpected because we believed that use of the shorter alkyl chain, smaller micelle detergents would make the MdtM protein-detergent complexes more amenable to crystallization due to availability of better protein-protein contacts for lattice formation. A major factor in the lack of crystallizability of these particular protein-detergent complexes under the conditions tested may be the observed ~10 °C decrease in thermostability of MdtM (as judged from the experimentally determined *T*_m_ values) when it was solubilized in UDM or DM compared to when the protein was purified using the modified protocol in DDM. The fact that MdtM-DDM protein-detergent complex prepared using the unmodified protocol was also refractory to crystallization under the conditions tested, and was significantly less thermostable than MdtM-DDM complexes purified using the modified protocol, highlights the influence of protein thermostability as an important variable in membrane protein crystallization [53].

Taken together, the results of the crystallization screens suggest that MdtM-DDM protein-detergent complexes produced using the modified purification method described in this work can be crystallized in three-dimensions. The crystallization conditions are currently being optimized to produce the diffraction-grade crystals necessary for successful elucidation of the 3D structure of MdtM by X-ray crystallography.

## 3. Experimental Section

### 3.1. Materials

All materials were purchased from Sigma-Aldrich (Poole, Dorset, UK) unless stated otherwise.

### 3.2. Cloning, Plasmids and Bacterial Strains

Cloning of the *E. coli mdtM* open reading frame into a modified pBAD/*Myc-*His A expression vector (Invitrogen, Paisley, UK) has been described previously [20]. Four additional histidine residues were introduced into the hexahistidine tag of the construct using QuikChange Multi site-directed mutagenesis (Agilent Technologies, Stockport, UK). The resulting DNA product was transformed into *E. coli* TOP10 cells (Invitrogen, Paisley, UK), isolated, and then verified by DNA sequence analysis. Sequence-verified plasmid DNA was transformed into *E. coli* LMG194 [F^−^ Δ*lac*X74 *gal*E *thi* *rps*L Δ*pho*A (*Pvu* II) Δ*ara*714 *leu*::Tn*10*] for overproduction of the protein.

### 3.3. Overproduction of MdtM and Membrane Preparation

A culture inoculated with a single colony of *E. coli* LMG194 transformed with pBAD/*Myc-*His A expression vector containing *mdtM* was grown overnight in Luria Bertani (LB) broth to an OD_600_ of 3.0 then diluted 200-fold into six 5 L flasks, each containing 1 L of LB broth supplemented with 100 μg/mL carbenicillin. These cultures were grown at 32 °C for 2 h with 220 rpm shaking prior to shifting the temperature to 25 °C. At OD_600_ of 0.8, recombinant MdtM expression was induced by addition of 6.7 mM L-(+) arabinose. Cells were grown for a further 2 h at 32 °C with 220 rpm shaking before harvesting by low speed centrifugation (5000× *g* for 15 min at 4 °C).

Cell pellets were resuspended in 50 mM Tris-HCl, pH 7.5, 100 mM NaCl containing 1 mM phenylmethanesulfonyl fluoride (PMSF), a protease inhibitor cocktail tablet (Roche Diagnostics, Burgess Hill, UK) and 5 μM DNase. The cell suspension was stirred for 30 min then passed three times through a pre-chilled French pressure cell at 1000 psi to break the cells. Unbroken cells and cell debris were removed by centrifugation at 18,000× *g* for 20 min. Cell membranes were collected by ultracentrifugation at 100,000× *g* for 2.5 h, resuspended in a membrane solubilisation buffer (20 mM Tris-HCl, pH 8.0, 500 mM NaCl, 10 mM imidazole, 10% v/v glycerol) that contained 1 mM PMSF and a protease inhibitor cocktail tablet, then homogenized and solubilised by addition of *n*-dodecyl β-d-maltoside (DDM; Melford Laboratories Ltd., Ipswich, UK) to ~25 mM and stirred for 1 h. The solubilized membrane fraction was subjected to high-speed centrifugation (100,000× *g*) for 30 min to remove any unsolubilised material. All the above and subsequent purification steps were performed at 4 °C.

### 3.4. Immobilized Metal Affinity Chromatography

The amount of 1.5 mL of 50% (w/v) Ni-NTA agarose resin (Qiagen, Manchester, UK) was added to a gravity-flow Kontes column, washed with water then incubated at 4 °C with membrane solubilisation buffer buffer (20 mM Tris-HCl pH 8.0, 500 mM NaCl, 10 mM imidazole, 10% v/v glycerol) for 30 min. DDM-solubilized membrane protein was applied to the Ni-NTA resin and the decahistidine-tagged MdtM was allowed to bind to the chromatography resin. The column was subsequently washed with 20 column volumes (CVs) of wash buffer (50 mM Tris-HCl pH 8.0, 100 mM NaCl, 20% v/v glycerol, 2.0 mM DDM, 30 mM imidazole) to remove non-specifically bound protein. Importantly, contaminating chaperonin was removed by incubating the resin with ATP dissociation buffer (wash buffer containing 10 mM MgCl_2_, 5 mM ATP, 150 mM KCl) at 4 °C for 2 h [37], followed by an additional wash with wash buffer. In contrast to the step-gradient elution with 3 mL of wash buffer containing 150 mM, 250 mM and 500 mM imidazole that was used previously to elute MdtM from the column [20], the optimized protocol employed a single elution step with 250 mM imidazole. Total protein in the eluate fractions were quantified by Micro BCA assay (Thermo Scientific Pierce, Paisley, UK) and protein fractions were analysed for purity by incubation with SDS-PAGE loading buffer for 10 min at room temperature and loading onto a 10% acrylamide SDS-PAGE gel. MdtM-containing fractions were pooled and 6 NIH units of thrombin/mg of protein added to remove the decahistidine affinity tag. The protein solution was then dialyzed overnight at 4 °C against 50 mM Tris-HCl pH 7.2, 300 mM NaCl, 0.5 mM DDM and 5% v/v glycerol using Snakeskin 10 kDa MWCO dialysis membrane (Pierce Thermo Scientific, Paisley, UK) to reduce the imidazole, glycerol and DDM concentrations.

### 3.5. Size Exclusion Chromatography

Dialyzed, thrombin-digested MdtM was concentrated to a volume of 1 mL using a 100 kDa MWCO Centricon centrifugal concentrator (Millipore, Livingston, UK). The concentrated protein sample was immediately loaded onto a Superdex-200 10/300 GL gel filtration column (GE Healthcare, Little Chalfont, UK) connected to an ÅKTA FPLC system and equilibrated with SEC buffer (50 mM Tris-HCl pH 7.2, 300 mM NaCl, 50 mM imidazole, 5% v/v glycerol, 0.25 mM DDM) supplemented with 5 mM dithiothreitol reducing agent immediately prior to injection. The inclusion of imidazole in the buffer was necessary to maintain stability of the protein during the SEC run. The same protocol and buffer system was used for purification of protein in undecylmaltoside (UDM) and decylmaltoside (DM) detergents, except that DDM was replaced with 0.6 mM UDM or 1.8 mM DM. Detergents for use in size exclusion chromatography were of high-purity (Anagrade), and supplied by Anatrace (Maumee, OH, USA). Chromatography runs were performed at a flowrate of 0.3 mL/min, and protein was monitored by absorbance at 280 nm. Peak protein fractions were collected and quantified by Micro BCA assay and protein purity was analysed by SDS-PAGE.

### 3.6. Detergent Screening

The stability and monodispersity of MdtM in a panel of seven detergents of different chain lengths and/or micelle size was assessed by FPLC-SEC using a Superdex-200 10/300 GL column (GE Healthcare). Tag-cleaved protein from the IMAC column was aliquoted into seven 1 mL fractions, each containing approximately 0.3 mg of protein, and and each was loaded onto the SEC column equilibrated with SEC buffer containing either 25 mM *n*-octyl-β-d-thioglucoside (OTG), 6.5 mM *n*-nonyl-β-d-glucoside (NG), 1.8 mM decylmaltoside (DM), 0.6 mM undecylmaltoside (UDM), 0.25 mM *n*-dodecyl β-d-maltoside (DDM), 2.0 mM *n*-dodecyl-*N*,*N*-dimethylamine-*N*-oxide (LDAO) or 1.5 mM *n*-dodecyl-*N*,*N*-dimethylglycine (DDMG). Chromatography runs were performed at a flowrate of 0.3 mL/min, and protein was monitored by absorbance at 280 nm. A 20 μL sample from the peak protein fraction of each run was analysed by 15% acrylamide SDS-PAGE.

### 3.7. Substrate Binding Assays

The affinity of purified MdtM for chloramphenicol substrate was assessed by intrinsic tryptophan fluorescence quenching studies. Briefly, fluorescence experiments were performed using a Fluoromax-4 fluorometer (Horiba, Stanmore, UK) by exciting exclusively into tryptophan residues, with excitation and emission wavelengths of 295 nm and 335 nm, respectively. Size exclusion-purified protein was dialyzed for 1 h at 4 °C against 50 mM Tris-HCl, pH 7.2, 100 mM NaCl, 5% v/v glycerol containing either 0.25 mM DDM, 0.6 mM UDM or 1.8 mM DM to remove imidazole and decrease NaCl concentration using GeBAflex mini dialysis tubes with a cut-off of 25 kDa (Molecular Dimensions, Newmarket, UK), then brought to a final concentration of 0.22 µM. The sample was titrated with chloramphenicol until saturation of the fluorescence quench at the fluorescence emission maximum of 335 nm was achieved. The fluorescence intensities were corrected for any inner filter effects as described in [54]. All experiments were performed in triplicate at 25 °C.

### 3.8. Thermostability Assays

The thermostability of MdtM was determined by measuring the change in intrinsic tryptophan fluorescence intensity as a function of temperature. Purified MdtM was dialyzed against buffer consisting of 50 mM Tris-HCl, pH 7.2, 100 mM NaCl, 5% v/v glycerol containing either 0.25 mM DDM, 0.6 mM UDM or 1.8 mM DM and the protein was brought to a final concentration of 0.22 µM in a 1.5 mL quartz cuvette. The cuvette was placed in a thermostatically-controlled cuvette holder in a Fluoromax-4 fluorometer and the contents stirred constantly. Fluorescence measurements were performed at an excitation wavelength of 295 nm, emission was monitored from 310–400 nm and the fluorescence intensity at the emission maximum of 335 nm recorded. Thermal denaturation was monitored by increasing the temperature in 5 °C increments and allowing the sample to equilibrate at each temperature for 2 min prior to recording the fluorescence intensity. Temperature-dependent denaturation of MdtM was determined using the formula [55]:

(1) $$f_{U}=\frac{\left(F_{F}-F_{O}\right)}{\left(F_{F}-F_{U}\right)}$$

where *f*_U_ is the fraction of unfolded protein, *F*_F_ is the fluorescence intensity of the completely folded fraction of protein, *F*_O_ is the observed fluorescence at any given temperature and *F*_U_ is the fluorescence intensity of the completely unfolded protein. Experiments were repeated in triplicate and the data were fitted using a Boltzmann sigmoidal curve in SigmaPlot 10 (Systat Software Inc., Hounslow, UK).

### 3.9. Mass Spectrometry

Protein for mass spectrometry analysis was prepared using the unmodified purification protocol. MdtM-containing fractions eluted from the IMAC column were separated on an SDS-PAGE gel of 15% acrylamide and the gel was subsequently stained with Coomassie Blue (see Figure 1a). The contaminating ~58 kDa band was excised, subjected to enzymatic digest using trypsin, then analysed by MALDI TOF/TOF mass spectrometry by the BMS Mass Spectrometry and Proteomics Facility, University of St. Andrews, UK.

### 3.10. Dynamic Light Scattering

The oligomeric state of MdtM in the detergents DDM, UDM and DM was determined by dynamic light scattering (DLS) using SEC-purified protein at concentrations of 1.0, 2.0 and 4.0 mg/mL. Each experiment was run in a disposable polystyrene cuvette at 25 °C for 60 s in a Zetasizer Nano (Malvern Instruments, Malvern, UK), using a laser with λ = 633 nm and a scattering angle of 173°. The oligomeric state of MdtM in DDM detergent was further verified by BN-PAGE gel analysis.

### 3.11. Blue Native PAGE

SEC-purified MdtM in DDM detergent solution was quantitated by Micro BCA assay. Protein was prepared for BN-PAGE by dilution with 3× gel buffer (750 mM aminocaproic acid, 150 mM Bis-Tris, pH 7.0 and 1.0 mM DDM) to give samples containing 33 µg, 11 µg, 3.7 µg, 1.2 µg or 0.4 µg MdtM protein in a final volume of 40 µL. Sample loading buffer (750 mM aminocaproic acid, 75 mM Bis-Tris/HCl, pH 7.0, 0.5mM EDTA, 5% w/v Serva Blue G) was added to a final buffer:sample ratio of 1:10 and samples were maintained on ice until use. All subsequent steps were performed at 4 °C. Samples were loaded onto a 3%–18% acrylamide gradient gel and run in cathode Buffer A (50 mM Tricine, 15 mM Bis-Tris, pH 7.0, 0.02% w/v Serva Blue G) at 30 V for the first 30 min. The voltage was increased to 80 V until the dye front reached the middle of gel, at which point cathode Buffer A was replaced with cathode Buffer B (50 mM Tricine, 15 mM Bis-Tris, pH 7.0). The gel was then run at 80 V until the dye front reached the end of the gel. Protein bands were visualized by silver staining.

### 3.12. Crystallization of MdtM

Peak fractions of MdtM from SEC in DDM, UDM or DM detergent were quantitated by Micro BCA assay and fractions containing a protein concentration greater than 1.5 mg/mL were pooled and concentrated to 10 mg/mL using a Centricon centrifugal device with a 100 kDa MWCO membrane (Millipore, Livingston, UK). Protein was dialyzed for 1 h at 4 °C using GeBAflex 25 kDa MWCO mini dialysis tubes (Molecular Dimensions, Newmarket, UK) against a buffer consisting of 50 mM Tris-HCl, pH 7.2, 10 mM NaCl and 10% v/v glycerol to reduce both detergent and NaCl concentrations. Crystallization screens were performed using the MemGold, MemStart, MemSys and PGA commercial screening kits from Molecular Dimensions, and the MemFac, Crystal Screen and Crystal Screen Lite kits from Hampton Research (Aliso Viejo, CA, USA) using the hanging drop vapour diffusion method. Trials were carried out by mixing 1 µL of protein with 1 µL of crystallization buffer on a glass cover slip and the mixture was allowed to equilibrate with a final reservoir volume of 500 µL buffer solution. Trials were carried out manually in 24-well XRL plates (Molecular Dimensions) and incubated at 18 °C in a vibration-free incubator. Plates underwent ocular inspection every two days during the first two weeks, followed up by weekly inspections.

## 4. Conclusions

Elucidation of the structures of membrane proteins that function in antimicrobial efflux is of hallmark importance for a more thorough understanding of the structural basis for multiple drug resistance and may, in the future, provide an avenue for development of novel antimicrobial compounds to combat multidrug resistant bacterial pathogens [56]. We contend that the purification protocol described here could contribute to this aspiration by being of general use for the production of the high-quality protein needed for 3D crystallization studies of other multidrug efflux members of the MFS.
